# National and subnational mortality effects of metabolic risk factors and smoking in Iran: a comparative risk assessment

**DOI:** 10.1186/1478-7954-9-55

**Published:** 2011-10-11

**Authors:** Farshad Farzadfar, Goodarz Danaei, Hengameh Namdaritabar, Julie Knoll Rajaratnam, Jacob R Marcus, Ardeshir Khosravi, Siamak Alikhani, Christopher JL Murray, Majid Ezzati

**Affiliations:** 1 Diabetes Research Center, Tehran University of Medical Sciences, Tehran, Iran; 2 Endocrinology and Metabolism Research Center, Tehran University of Medical Sciences, Tehran, Iran; 3 Department of Global Health and Population, Harvard School of Public Health, Boston, USA; 4 Ministry of Health and Medical Education, Tehran, Iran; 5 Institute for Health Metrics and Evaluation, University of Washington, Seattle, USA; 6 Department of Epidemiology and Biostatistics, School of Public Health, Imperial College, London, UK; 7 MRC-HPA Center for Environment and Health, Imperial College, London, UK

## Abstract

**Background:**

Mortality from cardiovascular and other chronic diseases has increased in Iran. Our aim was to estimate the effects of smoking and high systolic blood pressure (SBP), fasting plasma glucose (FPG), total cholesterol (TC), and high body mass index (BMI) on mortality and life expectancy, nationally and subnationally, using representative data and comparable methods.

**Methods:**

We used data from the Non-Communicable Disease Surveillance Survey to estimate means and standard deviations for the metabolic risk factors, nationally and by region. Lung cancer mortality was used to measure cumulative exposure to smoking. We used data from the death registration system to estimate age-, sex-, and disease-specific numbers of deaths in 2005, adjusted for incompleteness using demographic methods. We used systematic reviews and meta-analyses of epidemiologic studies to obtain the effect of risk factors on disease-specific mortality. We estimated deaths and life expectancy loss attributable to risk factors using the comparative risk assessment framework.

**Results:**

In 2005, high SBP was responsible for 41,000 (95% uncertainty interval: 38,000, 44,000) deaths in men and 39,000 (36,000, 42,000) deaths in women in Iran. High FPG, BMI, and TC were responsible for about one-third to one-half of deaths attributable to SBP in men and/or women. Smoking was responsible for 9,000 deaths among men and 2,000 among women. If SBP were reduced to optimal levels, life expectancy at birth would increase by 3.2 years (2.6, 3.9) and 4.1 years (3.2, 4.9) in men and women, respectively; the life expectancy gains ranged from 1.1 to 1.8 years for TC, BMI, and FPG. SBP was also responsible for the largest number of deaths in every region, with age-standardized attributable mortality ranging from 257 to 333 deaths per 100,000 adults in different regions.

**Discussion:**

Management of blood pressure through diet, lifestyle, and pharmacological interventions should be a priority in Iran. Interventions for other metabolic risk factors and smoking can also improve population health.

## Introduction

Iran has experienced unprecedented demographic changes over the past few decades. According to the General Population and Housing Census, the number of adults older than 64 years of age increased from 1.2 million in 1976 (3.7% of the population) to 3.5 million in 2006 (5.5% of the population). Chronic diseases, especially cardiovascular diseases (CVD), have also become a more prominent cause of death, responsible for 47% of all deaths in 1995, compared with 27% in 1981 [1].

An important question for planning and allocating resources is the role of metabolic and lifestyle risk factors on mortality, especially from CVD. A previous analysis [2] estimated the mortality effects of major risk factors in Iran but was based on data that were not nationally representative. Further, the previous analysis estimated the effects of systolic blood pressure (SBP), total cholesterol (TC), and body mass index (BMI) below and above clinical thresholds, even though epidemiologic studies have shown that the association between these risk factors and CVD risk continues below such thresholds [3,4]. Additionally, there has not been an assessment of the incompleteness of the vital registration system and misclassification of cause of death, or of uncertainty of the estimated number of deaths attributable to risk factors. Nationally and provincially representative data on risk factors are now available for 2005 from a health examination survey focused on noncommunicable diseases, and large meta-analyses of cohorts have provided new evidence on the effects of these risk factors on CVD [4,5]. There are also new methods that help adjust for incompleteness of mortality registration [6].

We used these advances in data and methods to estimate the mortality effects of metabolic and lifestyle risk factors at the national level. To understand how risk factors may affect mortality in different parts of Iran, we extended the analysis to groups of provinces defined by geography and socioeconomic status. We also quantified the uncertainty in our results.

## Methods

We conducted a population-level comparative risk assessment (CRA) for five modifiable risk factors. Based on data availability on exposure and effect size, the risk factors in the analysis were smoking and four metabolic risks: BMI, SBP, fasting plasma glucose (FPG), and TC. The CRA analysis estimates the number of deaths that would have been prevented if past and current exposure to risk factors were reduced to a hypothetical alternative distribution. The inputs to the analysis are: [1] the current distribution of exposure to the risk factor in the population, [2] the etiological effect of the risk factor on disease-specific mortality, [3] an alternative exposure distribution, and [4] the total number of disease-specific deaths in the population. Analyses were done separately by sex and by the following age groups: 30-44, 45-59, 60-69, 70-79, and ≥ 80 years.

### Exposure to risk factors

Means and standard deviations (SD) for the metabolic risk factors were estimated from the 2005 Non-Communicable Disease Surveillance Survey (NCDSS), which is representative at the national and provincial level, and uses a multistage systematic clustered sampling applied to rural and urban areas of all provinces [7,8]; the survey was implemented simultaneously in all provinces between December 2004 and February 2005 and included 89,400 individuals aged 15 to 64 years. The survey included a questionnaire and physical and laboratory measurements of weight, height, and systolic and diastolic blood pressure for all participants and FPG and lipids for 50,202 participants aged 25 to 64 years old. SBP was measured using a standard mercury sphygmomanometer, with each participant measured three times in sitting position; weight was measured using a calibrated beam scale; and plasma was separated from the fasting blood sample and analyzed on the same day.

We imputed the mean and SD of metabolic risks in age groups above 65 years of age using data from 47 health examination surveys from other countries. Specifically, we estimated the linear slope between the ages of 30 and 64 by province for the NCDSS and for data from 47 health examination surveys from other countries with data for ages 65 years and older. We divided the slopes between 30 and 64 years from the latter surveys into tertiles; for each tertile, we estimated the average slope < 65 years in data pooled from all surveys, adjusting for inter-survey differences (i.e., survey-specific intercepts). For each Iranian province, the slope of each risk factor by age for ages 65 and older was taken from the tertile to which its slope below age 65 years belonged. We estimated the SD of the distribution for each risk factor for age groups above 65 years using the SD-mean relationship across all 47 health examination surveys. Following previous analyses [9-11] we used lung cancer mortality to measure cumulative population exposure to smoking.

One-off measurement of metabolic risk factors in health examination surveys overestimates the SD of the "usual" population exposure distribution, due to within-person variation. We estimated the usual population SD of SBP, FPG, and TC by multiplying the SD of the NCDSS sample by the dilution ratio from studies that had multiple exposure measurements [12-14]. We did not adjust the SD of BMI for within-person variations in body weight, because studies with multiple BMI measurements have not found evidence for substantial within-person variability in BMI [15].

### Etiologic effects of risk factors

For each risk factor-disease pair with convincing evidence of causality, relative risks (RRs) were derived from recent reviews of epidemiologic studies, summarized in the United States CRA study [10]. Previous work has shown that after accounting for study design and methodological differences, etiological effect sizes are consistent across different populations [16], and hence, in the absence of reliable evidence for etiological effects from Iran, large meta-analyses of international cohorts are the best source of etiological effect sizes.

### Disease-specific mortality

Population data by age, sex, and province were from the 1996 and 2006 national censuses. Deaths by age, sex, province, and underlying cause of death were from the mortality registration system, which covers all 30 provinces but excludes Tehran city, where 13.5% of the population lives [17]. We used data from Tehran's central cemetery for Tehran city. To examine and correct the incompleteness of death registration, we used death distribution methods [6,18]. Specifically, we evaluated three methods: Generalized Growth Balance (GGB), Synthetic Extinct Generations (SEG), and a hybrid of the two methods (GGBSEG). We chose SEG because it gave the highest correlation between the estimated adult and child mortality completeness (*r_SEG _*= 0.16, *r_GGB _*= 0.04, *r_GGBSEG _*= 0.035). We estimated child mortality and completeness of child death registration by comparing under-5 mortality estimates from the death registration system to estimates generated from the 2001 Demographic and Health Survey (DHS) and the 2006 census, an approach that has been extensively used in the literature [19].

Of the 262,000 deaths in the Iranian death registration system in 2005, about 19,000 deaths (7.4%) had an unknown cause. We used data from a study in 2005 that reviewed hospital medical records by trained physicians [20] to estimate the underlying medical causes for these deaths. We applied multinomial logistic regression to data from the above study and used the regression coefficients to estimate the proportions of different diseases as underlying causes for these deaths, as a function of the decedent's age, sex, and province of residence. Age was missing for 0.7% and sex for 0.4% of deaths. We used multiple imputation using Amelia software [21] to impute missing age and sex values.

### Mortality effects of risk factors

For each risk factor and for each disease causally linked with its exposure, we computed the proportional reduction in disease-specific deaths that would occur if risk factor exposure had been reduced to an alternative level, known as the population-attributable fraction (PAF). For risks measured continuously (SBP, BMI, TC, and FPG), we computed PAFs using equation 1:

(1) $$PAF=\frac{\overset{m}{\underset{x=0}{\int }}RR\left(x\right)P\left(x\right)\;dx-\overset{m}{\underset{x=0}{\int }}RR\left(x\right)P^{\prime }\left(x\right)\;dx}{\overset{m}{\underset{x=0}{\int }}RR\left(x\right)P\left(x\right)\;dx}$$

where *x *is the risk factor exposure level, *P*(*x*) is the actual exposure distribution, *P'*(*x*) is the alternative exposure distribution, *RR*(*x*) is the relative risk of mortality at exposure level *x *for that specific disease, and *m *is the maximum exposure level.

We used the theoretical minimum risk exposure distribution (TMRED) as the alternative exposure distribution to measure the mortality effects of all nonoptimal levels of exposure consistently and comparably across risk factors [10,22]. Based on a previous review of epidemiological evidence, the mean (g ± SD) of the TMREDs was chosen to be 21 (± 1) kg/m^2 ^for BMI, 115 (± 6) mmHg for SBP, 3.8 (± 0.6) mmol/L for TC, and 4.9 (± 0.3) mmol/L for FPG.

We calculated deaths, mortality rates, and loss of life expectancy attributable to each risk factor. The mortality rates were age standardized using the national Iranian population in 2005 as the reference population. Life expectancies were calculated using standard life-table methods and accounted for the effects of death rates in older ages using the Coale-Guo method [23,24]. Life expectancy under the TMRED distribution was calculated by reducing mortality rates in each life table age group by the proportion attributable to risk factors and recalculating the life tables.

### Uncertainty in estimates

We quantified the uncertainty of the number of deaths attributable to each risk factor, accounting for uncertainty due to sampling variability of the exposure data and those of other input parameters. We used a simulation approach to combine the uncertainties of exposure distributions, RRs, and disease-specific mortality in each age-sex group. Under 65 years of age, in each iteration, we used one randomly-drawn exposure mean and SD from their distributions (normal and Chi-square distributions, respectively). Above 65 years, the uncertainty of the coefficients of the age-association regression model was also added using repeated draws from their distributions. RRs for each disease were drawn from a log-normal distribution, independently from exposure draws. Each set of exposure and disease-specific RR draws was used to calculate one PAF, separately by age group and sex. We expressed the uncertainty related to incompleteness of death registration as variance of the estimated level of completeness, assuming a SD of 20% of the estimated completeness [6]. We used 1,000 draws for each of the above parameters in repeated calculations, and reported 95% uncertainty intervals (UIs) based on the resulting distributions of 1,000 estimated attributable deaths. The above analysis does not include the uncertainty associated with the basic assumptions regarding the extrapolation of age patterns and RRs across populations.

### Subnational analysis

**Figure 1:**
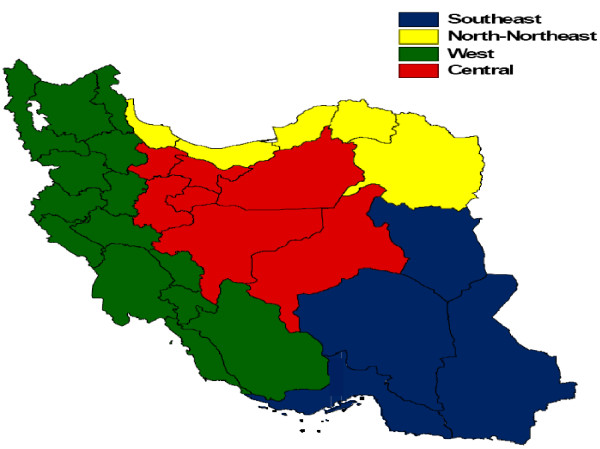
**Geographic regions used in the analysis**.

We conducted analyses by province and also by four regions. The regions were defined based on a combination of geography and socioeconomic status (SES). SES was measured using an index constructed from variables from the 2006 census, including years of schooling, employment rates, and family assets [25]. We combined these characteristics using principal component analysis and used these principal components in combination with geography to divide the country into four large regions (Figure 1). The Southeast region has the lowest SES and the Central region the highest.

All analyses were done using R version 2.11.1.

## Results

### Mortality and life expectancy in Iran

After correcting for incompleteness, there were an estimated 352,000 deaths in Iran in 2005. Of these, 277,000 deaths occurred in adults 30 years of age or older (59% in men). 53% of deaths above age 30 were due to CVD, including 26% from ischemic heart disease (IHD), 13% from stroke, 4% from hypertensive diseases, and 10% from other CVD. Life expectancy at birth in Iran was 70.0 years for men, ranging from 68.2 years in the Western region to 70.5 years in the Southeast; for women, it was 74.6 years and ranged from 73.5 years in the Western region to 75.6 years in the Southeast.

### Risk factor exposure

**Figure 2:**
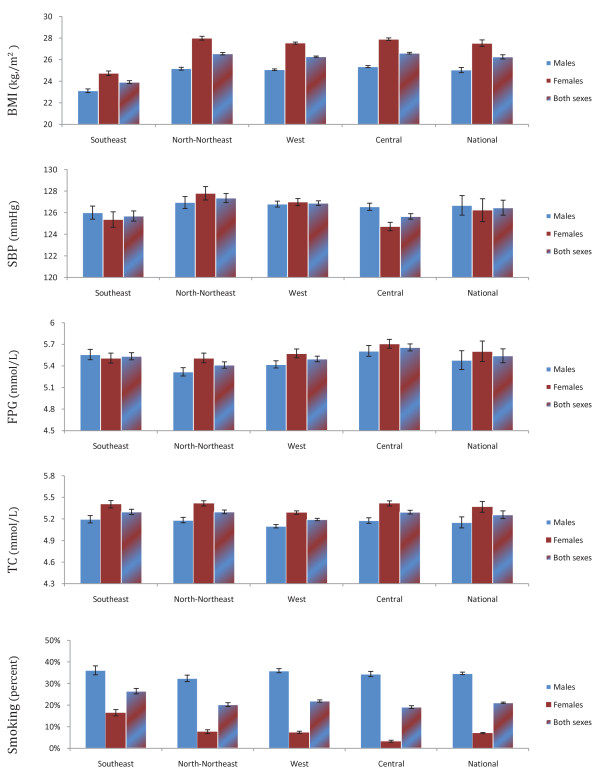
**Prevalence of smoking and mean levels of other risk factors in 2005, by region and sex**. All figures are age-standardized to the 2005 national population.

Estimated mean SBP was similar between men (126.7 mmHg; 95% UI: 126.5, 127.9) and women (126.2 mmHg; 126.0, 126.5) (Figure 2). Women had higher BMI (27.5 kg/m^2^; 27.5, 27.6), FPG (5.60 mmol/L; 5.56, 5.64), and TC (5.37 mmol/L; 5.35, 5.38) than men, while men had a higher prevalence of smoking (34.7%; 34.0, 35.4) than women (6.7%; 6.4, 7.0) at the national level (Figure 2). Age-standardized mean BMI and TC for both sexes and SBP for women in Iran were higher than the mean global level [26-28].

The differences across regions in SBP were not statistically significant, although SBP tended to be lowest in the Central region and highest in the North-Northeast region. The Central region, with the highest SES, had the highest FPG (5.61 mmol/L; 5.53, 5.68). The Southeast region, with the lowest SES, had the highest prevalence of smoking (36.1% for men and 16.4% for women) but the lowest BMI (23.1 kg/m^2 ^for men and 24.7 kg/m^2 ^for women).

### Mortality effects of risk factors

**Figure 3:**
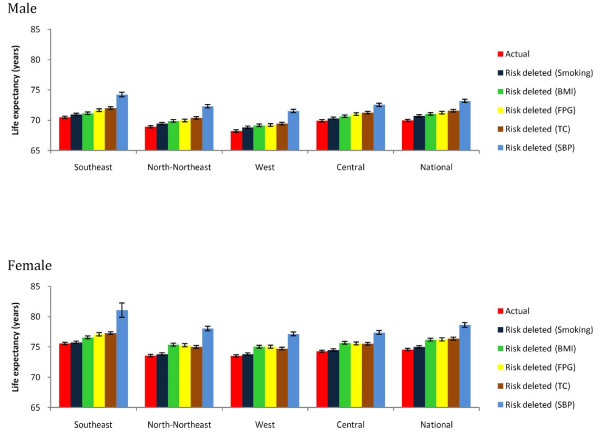
**Effects of each risk factor on life expectancy at birth in 2005, by region and sex**.

SBP was the leading risk factor for cardiovascular mortality, causing 41,000 deaths (38,000, 44,000) among men and 39,000 deaths (36,000, 42,000) among women (Table 1). If SBP is lowered to its optimal distribution, life expectancy at birth would increase by 3.2 years (2.6, 3.9) in men and 4.1 years (3.2, 4.9) in women (Figure 3). The age-standardized mortality attributable to SBP ranged from 257 to 333 deaths per 100,000 adults among men and women in different regions. TC and FPG had the second-largest effect on mortality among men, each causing 17,000 to 18,000 deaths, less than half of the deaths due to increased SBP. Among women, SBP was followed by FPG and BMI, each responsible for about 17,000 deaths. Life expectancy effects for FPG, BMI, and TC ranged from 1.1 to 1.8 years in both sexes. Smoking had the smallest mortality effects for both men (9,000 deaths, about one-fifth of the number attributable to SBP) and women (2,000 deaths, < 5% of the number attributable to SBP).

**Table 1:** Number of deaths attributable to each risk factor in 2005, by region and sex

	BMI		FPG		SBP		TC		Smoking	
	
	Male	Female	Male	Female	Male	Female	Male	Female	Male	Female
	
	Mean (95% UI)	Mean (95% UI)	Mean (95% UI)	Mean (95% UI)	Mean (95% UI)	Mean (95% UI)	Mean (95% UI)	Mean (95% UI)	Mean (95% UI)	Mean (95% UI)
National	13000 (11000, 15000)	17000 (14000, 20000)	17000 (14000, 20000)	17000 (14000, 20000)	41000 (38000, 44000)	39000 (36000, 42000)	18000 (16000, 20000)	16000 (14000, 18000	9000 (8600, 9400)	2000 (1900, 2100)

Southeast	700 (600, 800)	700 (600, 800)	1400 (1100, 1700)	1100 (900, 1300)	3200 (3100, 3300)	2800 (2700, 2900)	1500 (1400, 1600)	1200 (1100, 1300)	500 (490, 510)	200 (190, 210)

North-Northeast	3200 (2700, 3700)	4400 (3700, 5100)	3200 (2600, 3800)	3900 (3200, 4600)	8800 (8400, 9200)	9100 (8700, 9500)	4200 (3900, 4500)	3800 (3500, 4100)	1600 (1560, 1640)	500 (480, 520)

West	5400 (4600, 6200)	7100 (5900, 8300)	6500 (5300, 7700)	6300 (5100, 7500)	16800 (16300, 17300)	15100 (14700, 15500)	7000 (6700, 7300)	5800 (5500, 6100)	3900 (3800, 4000)	800 (780, 820)

Central	3700 (3200, 4200)	5300 (4300, 6300)	5300 (4400, 6200)	5300 (4300, 6300)	12600 (12100, 13100)	11900 (11400, 12400)	5700 (5300, 6100)	4600 (4200, 5000)	2600 (2500, 2700)	900 (870, 930)

The PAFs for the effects of all four metabolic risk factors on IHD and stroke were larger in women than men, due to higher exposure. However, deaths attributable to these risks were larger among men, due to the larger number of CVD deaths. Reducing these risk factors to their optimal levels would have led to gains in life expectancy that were nonsignificantly larger in women, possibly due to larger PAFs and differences in distributions of deaths by age and competing causes.

**Figure 4:**
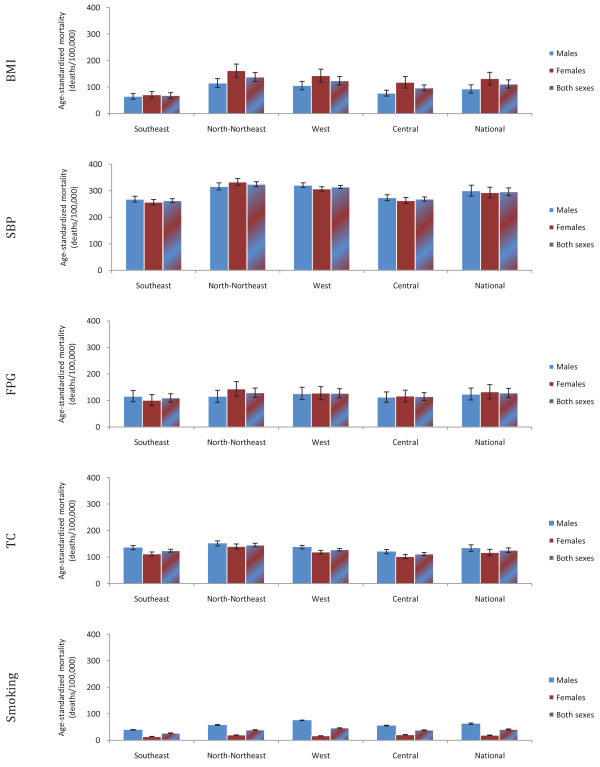
**Age-standardized mortality rates attributable to each risk factor in 2005, by region and sex**.

High SBP was the leading cause of mortality in all regions. Age-standardized mortality rates attributable to SBP were highest in the North-Northeast and Western regions, ranging from 310 to 330 deaths per 100,000 adults for both sexes; they were lowest in the Southeast in both sexes, because the age-standardized cause-specific mortality rate from CVD is lowest in this region (Figure 4). The North-Northeast and Western regions also had the largest PAF for the effects of SBP on IHD and stroke, ranging from 45% to 63% (detailed results not shown). However, the effects of SBP on life expectancy were largest in the Southeast (3.8 years gained in men and 5.5 years gained in women), possibly because CVD deaths occur in younger ages in this region.

Although the Southeast had the highest reported prevalence of current smoking, age-standardized mortality attributable to smoking, calculated using lung cancer mortality as indicator of cumulative exposure, was largest in the Central region among women (21 deaths per 100,000; 20, 22) and the Western region among men (75 deaths per 100,000; 74, 76). The differences may be due to longer history of smoking in the Central and Western regions, as well as higher age-specific mortality rates from diseases caused by smoking.

The highest provincial age-standardized mortality rate attributable to SBP was about 70% larger than the lowest one (the difference was about 150 deaths per 100,000). For BMI, FPG, and TC, the highest attributable mortality rate was 1.2 to 2.2 times that of the lowest. For smoking, the highest attributable mortality rate was 12 times higher in women and 32 times higher in men than that of the lowest.

## Discussion

Our results indicate that high SBP had the single largest mortality impact nationally, as well as in every province and region in Iran, causing an estimated 80,000 annual deaths in 2005. If SBP were reduced to optimal levels, life expectancy at birth would increase by 3.2 years (2.6, 3.9) and 4.1 years (3.2, 4.9) in men and women, respectively. High FPG, BMI, and TC were responsible for about one-third to one-half of deaths attributable to SBP in men and/or women. The effects of smoking on life expectancy at the national and subnational levels were less than one year, primarily because the rise in smoking is a more recent phenomenon in Iran than in Western countries and even East Asia.

Our analysis extends the emerging body of research on national and subnational CRA analyses [10,29-31]. The strengths of our study include combining national and subnational analyses using representative data, using effect sizes from large meta-analyses, estimating incompleteness of death registration using rigorous demographic methods, redistributing deaths with unknown causes, and quantification of uncertainty.

A key limitation of our study is that we did not have data on dietary factors, physical activity, alcohol and illicit drug use, or other metabolic risk factors, e.g., lipoproteins. Moreover, our exposure data did not include people 65 years of age and older, requiring extrapolation and leading to additional modeling assumptions and uncertainty beyond the quantified statistical uncertainty. In 2005, 5.5% of Iran's population and 49% of deaths were in those aged 65 years and older. We used RRs in specific cohorts and their meta-analyses. While this extrapolation adds a source of unquantifiable uncertainty, population-level estimation is indispensable to inform policymaking. More importantly, there is empirical evidence to support the proposition that proportional effects are similar across populations, e.g., Western and Asian populations [12,32,33]. Despite these limitations, our analysis, and those of others [10,29-31] demonstrate the value of nationally and subnationally representative data on risk factors for policy formulation and planning.

Our results have a number of implications for national and subnational health policies and programs in Iran, as well as for other middle-income countries. First, our analysis highlights the importance and the need for periodic risk factor surveillance studies to measure trends, which may be used for evaluating implemented policies. Recent comprehensive systematic reviews of metabolic risk factors showed that developing countries in Latin America and the Caribbean, Middle East and North Africa, Central and Southeast Asia, and sub-Saharan Africa had limited data, especially longitudinal data, on metabolic risk factors [26-28,34]. Our study supports the value of population-based surveillance for not only comparative cross-country analysis, but also for national and subnational priority setting. The demographic and epidemiologic transitions in Iran and other middle-income countries, which inevitably lead to aging of the population, make it essential to increase the participation of the elderly in health examination surveys. Future surveys should also include important dietary and lifestyle risk factors that were not included in our analysis.

Beyond risk factor surveillance, interventions are needed to address metabolic risk factors. Many countries have successfully lowered blood pressure levels in the past three decades; for example, blood pressure decreased by about 2.0 to 4.0 mmHg per decade in western European countries and Australia [28]. Although Iran has a hypertension control program, it only focuses on high-risk patients [35]. Our results indicate that a greater emphasis on reducing blood pressure at the population level through improved detection and better treatment in primary care is needed to reduce CVD mortality. Further, population-level analyses of trends and meta-analyses of randomized trials have shown that lowering salt intake can lower blood pressure [36,37]. Since the traditional Iranian diet is wheat-based [38], reducing salt in bread through regulation or large-scale media campaigns may help lower blood pressure at the population level.

There is currently no comprehensive program to control obesity in Iran, either in the primary health care system or through lifestyle and dietary interventions, although diabetes management has been integrated in the primary care system in rural areas [39]. Community-based interventions such as family-oriented advice on healthy eating and physical activity by a dietitian have been shown to increase physical activity, improve dietary habits, and reduce weight gain [40]. Integrating obesity and diabetes prevention and control programs in the primary health care system and using community health workers might help increase compliance and reduce these risk factors, as has been done for diabetes in rural areas [39]. Identifying, implementing, and evaluating such interventions are particularly important in the light of rising overweight and obesity in Iran and worldwide [27], which would inevitably lead to an increase in diabetes and hypertension unless population-based interventions are implemented.

## Competing interests

The authors declare that they have no competing interests.

## Authors' contributions

All authors have read and approved the final manuscript. Designed the study: FF, GD, and ME. Analyzed the data: FF and GD. Wrote the first draft of the paper: FF and ME. Contributed to the writing of the paper: GD, HN, JR, JM, AK, SA, CJLM. Supervised the research:ME.
